# A Machine Learning-Based Severity Prediction Tool for the Michigan Neuropathy Screening Instrument

**DOI:** 10.3390/diagnostics13020264

**Published:** 2023-01-11

**Authors:** Fahmida Haque, Mamun B. I. Reaz, Muhammad E. H. Chowdhury, Mohd Ibrahim bin Shapiai, Rayaz A. Malik, Mohammed Alhatou, Syoji Kobashi, Iffat Ara, Sawal H. M. Ali, Ahmad A. A. Bakar, Mohammad Arif Sobhan Bhuiyan

**Affiliations:** 1 Centre of Advanced Electronic and Communication Engineering, Department of Electrical, Electronic and Systems Engineering, Universiti Kebangsaan Malaysia, Bangi 43600, Malaysia; 2 Laboratory of Emotions Neurobiology, Nencki Institute of Experimental Biology, Polish Academy of Sciences, Ludwika Pasteura 3, 02-093 Warszawa, Poland; 3 Malaysia-Japan International Institute of Technology, Universiti Teknologi Malaysia, Jalan Sultan Yahya Petra, Kuala Lumpur 54100, Malaysia; 4 Department of Electrical Engineering, Qatar University, Doha 2713, Qatar; 5 Department of Medicine, Weill Cornell Medicine—Qatar, Doha 24144, Qatar; 6 Neuromuscular Division, Hamad General Hospital, Doha 3050, Qatar; 7 Department of Neurology, Al khor Hospital, Doha 3050, Qatar; 8 Graduate School of Engineering, University of Hyogo, Himeji 678-1297, Hyogo, Japan; 9 Electrical and Electronics Engineering, Xiamen University Malaysia, Sepang 43900, Malaysia

**Keywords:** DSPN, severity grading, nomogram, MNSI, machine learning

## Abstract

Diabetic sensorimotor polyneuropathy (DSPN) is a serious long-term complication of diabetes, which may lead to foot ulceration and amputation. Among the screening tools for DSPN, the Michigan neuropathy screening instrument (MNSI) is frequently deployed, but it lacks a straightforward rating of severity. A DSPN severity grading system has been built and simulated for the MNSI, utilizing longitudinal data captured over 19 years from the Epidemiology of Diabetes Interventions and Complications (EDIC) trial. Machine learning algorithms were used to establish the MNSI factors and patient outcomes to characterise the features with the best ability to detect DSPN severity. A nomogram based on multivariable logistic regression was designed, developed and validated. The extra tree model was applied to identify the top seven ranked MNSI features that identified DSPN, namely vibration perception (R), 10-gm filament, previous diabetic neuropathy, vibration perception (L), presence of callus, deformities and fissure. The nomogram’s area under the curve (AUC) was 0.9421 and 0.946 for the internal and external datasets, respectively. The probability of DSPN was predicted from the nomogram and a DSPN severity grading system for MNSI was created using the probability score. An independent dataset was used to validate the model’s performance. The patients were divided into four different severity levels, i.e., absent, mild, moderate, and severe, with cut-off values of 10.50, 12.70 and 15.00 for a DSPN probability of less than 50, 75 and 100%, respectively. We provide an easy-to-use, straightforward and reproducible approach to determine prognosis in patients with DSPN.

## 1. Introduction

Diabetic sensorimotor polyneuropathy (DSPN) leads to ulceration and amputation which are independently associated with increased mortality [1]. Early identification of DSPN is key to improve risk factors that may prevent the progression of DSPN [2,3,4,5]. The American Diabetic Association (ADA) [1] and Toronto consensus statements [6] recommended that the diagnosis of DSPN should be based on an assessment of symptoms and signs and nerve conduction studies (NCS). A number of diagnostic techniques are available for DSPN [1,7,8,9,10], alongside several composite scoring methods for severity stratification [11,12,13]. The Toronto consensus endorsed the use of a composite screening technique for defining the severity of DSPN [6].

The Michigan neuropathy screening instrument (MNSI) is a commonly utilized composite scoring technique recommended in the ADA position statement [1] for the clinical diagnosis of DSPN. It is a simple, inexpensive, reliable, and accurate assessment [13,14] that has been used to identify DSPN in many studies and clinical trials [14,15,16,17,18,19,20]. Neuropathy symptoms are assessed from 15 yes/no questions and neuropathy signs are assessed from five simple clinical tests. A patient is considered to have DSPN if the total score is ≥7 or ≥2 on the MNSI questionnaire or clinical tests, respectively [13]. However, there is controversy on the optimal cut-off value for identifying DSPN, with studies suggesting different cut-offs ranging from 2 to 1.5 [18], 2.5 [18,19,20], 3 [18] and 4 [21]. Moghtaderi et al. [18] reported an MNSI cut-off of 2 with a reliability of 0.81. Other studies have reported 80% sensitivity and 95% specificity and good repeatability for an MNSI examination cut-off ≥2.0 [13]. Herman et al. [19] suggested the use of MNSI in clinical trials due to its ease of use compared to NCS. However, the MNSI lacks a standardized grading system for severity classification.

Recently, machine learning (ML) approaches have been successfully used to solve different disease prediction and classification problems [22,23,24], because of their ability and reliability in extracting information from complex, non-linear, or incomplete data, supporting healthcare professionals in decision-making [25,26]. The fuzzy inference system (FIS) [27,28], multi-category support vector machine (SVM) learning [29], and adaptive fuzzy inference system (ANFIS) [30], have been reported to aid in the identification and stratification of diabetic neuropathy (DN). However, fuzzy systems-based classifiers do not appear to be reliable because they make use of the if-then rule-based set. Kazemi et al. [29], put forward a DSPN severity classifier based on a multiclass SVM, utilizing the neuropathy disability score (NDS), and reported an accuracy of 76%. Haque et al. [30] used ANFIS to report an accuracy of 91% for DSPN severity classification based on three MNSI variables (vibration perception, questionnaire, and tactile sensitivity). Reddy et al. [31] identified various risk factors for DN and proposed a Radial basis function (RBF) network for DN prediction, but only achieved 68.18% accuracy. Chen et al. [32] developed a prediction model to identify diabetic peripheral neuropathy (DPN) using MNSI by applying logistic regression (LR) and reported the value of the concordance index (c-index) to be 0.75.

We have deployed ML to develop a DSPN severity grading system from MNSI data. Initially, the most appropriate MNSI features were identified from a nomogram based on multivariable logistic regression and this was then developed and validated for classifying the severity of DSPN.

## 2. Materials and Methods

### 2.1. Database Description

Two different Michigan neuropathy screening instrument datasets were collected. The first dataset was sourced from the Epidemiology of Diabetes Interventions and Complications (EDIC) study [33,34]. In EDIC, the MNSI was used annually to assess DSPN in patients with type 1 diabetes [33,34]. A detailed description of the EDIC trial procedures and baseline characteristics of the patients have been reported previously [33,34,35].

Validation of our model was achieved in an independent MNSI dataset made available by Watari et al. [28] and is comprised of 102 patients with 21 MNSI variables: 15 questionnaires, vibration perception (L), vibration perception (R), 10-gm filament (combined results from both legs), the appearance of deformities (combined results from both legs), the appearance of callus (combined results from both legs), the appearance of fissure (combined results from both legs). For consistency we considered 21 variables from both data sets to design our prediction model.

### 2.2. Data Imputation

In practice, missing values in clinical data from larger clinical trials such as EDIC are quite a common phenomenon. Because the training of ML models depends highly on the dataset provided, missing data can be misleading for ML model training. To overcome this issue, data imputation techniques were applied [36]. MNSI data from 19 years of EDIC trials with 14,166 samples were collected. Many duplicate responses were removed, and 3754 unique samples were retrieved. In this study, missing data were calculated by the multiple imputations by chained equations (MICE) technique [37,38].

### 2.3. Feature Ranking

To ascertain the best possible combination of MNSI features to identify DSPN, three different feature ranking techniques, namely random forest (RF), [39] multi-tree extreme gradient boost (XGBoost) [40], and extremely randomized trees (extra tree) [41] techniques were used, and the best-performing algorithm was identified and reported. The in-house code for data imputation and feature ranking was written using Python 3.7.

### 2.4. Logistic Regression Classifiers

A supervised logistic regression classifier was utilized [42] for validating the performance of the top entries of the feature ranking. Logistic regression is commonly used for biomedical classification tasks [42,43], and in this case could assess the association of multiple variables with an outcome, e.g., DSPN or non-DSPN. The dataset was partitioned into a 70/30 ratio for the train and test set. The LR model was trained using five-fold cross-validation. Different performance parameters were calculated for evaluating the model’s performance.

### 2.5. Development and Validation of Logistic Regression-Based Nomogram

A diagnostic nomogram was constructed by Zlotnik and Abraira [44] using multivariate logistic regression analysis in Stata/MP software (StataCorp LLC, College Station, TX, USA). The multivariate logistic regression model was developed for two classes: DSPN and non-DSPN. The coefficients calculated from the LR model were used to calculate linear prediction as shown in Equations (1) and (2). Using Equation (2), we calculated the probability of having DSPN, as shown in Equation (3).

(1) $$coefficients=\frac{p}{1-p}$$

(2) $$Linear\;Prediction\;\left(LP\right)=\ln\left(\frac{p}{1-p}\right)$$

(3) $$p=\frac{e^{LP}}{1-e^{LP}}$$

The top-ranked features (i.e., the independent variables) exhibiting the best performance with the LR classifier were used to create the logistic regression-based nomogram. Calibration curves were plotted for evaluating the performance of the model. Utilizing the Stata tool, we also performed the decision curve analysis (DCA) for identifying the threshold values for clinically useful nomograms.

### 2.6. Development and Validation of Severity Grading Score

From the nomogram, a four-class DSPN severity scoring technique was proposed based on the probable cut-off values. The performance of the proposed grading system was validated with EDIC ground truth and the grading system proposed in [28].

## 3. Results

### 3.1. Patients’ Characteristics and Clinical Outcomes

**Figure 1:**
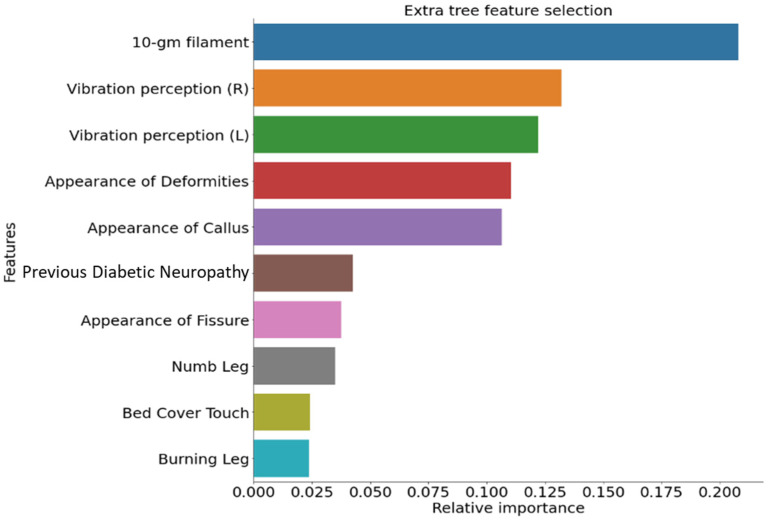
Top-10 ranked features identified using Extra Tree algorithms from the data imputed utilizing the MICE algorithm.

The EDIC patients’ baseline demographic variables are presented in Table 1. More details on EDIC patients can be found in other studies [33,34,35]. From the collected dataset, 3754 unique data samples were retrieved after removing duplicate responses. Among the 3754 unique samples, 2177 samples were from non-DSPN and the remaining 1577 samples were from DSPN patients. Figure 1 demonstrates the top-10 ranked MNSI features, as identified by the extra tree feature ranking technique. These are sensitivity to the 10-gm filament, vibration perception (L), vibration perception (R), the appearance of callus, appearance of deformities, previous diabetic neuropathy, the appearance of fissure, numb leg, burning leg, and response to bed cover touch. The results of the Xgboost and RF feature ranking techniques are shown in Supplementary Figures S1 and S2. There is no difference in the ranked features by the extra tree and RF technique. Therefore, we studied the extra tree and Xgboost technique to find the combination of features with the best performance.

### 3.2. Univariate Logistic Regression Model for Identifying Variables Significantly Associated with DSPN

**Figure 2:**
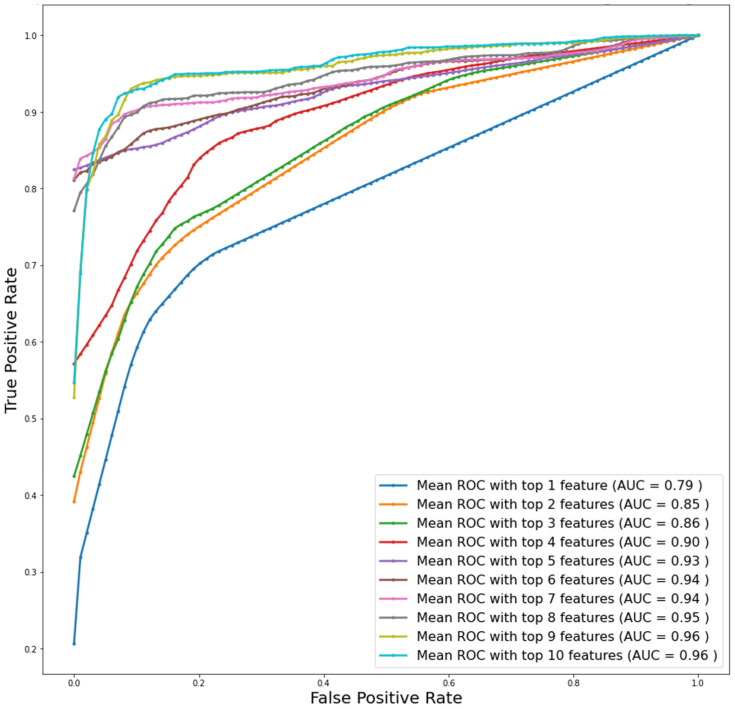
Receiver operating characteristic (ROC) plots for the top-10-ranked features utilizing the MICE data imputation and logistic regression classification techniques for extra tree feature selection algorithms.

Both the top 9 and top 10 features had an AUC of 0.96 for the data imputed utilizing MICE and the extra tree feature-ranking technique (Figure 2). Visually, it seems that model performance was saturated after the top 9 features. To confirm and identify the best possible combination of the features, we used logistic regression classifiers for performance evaluation. In order to determine how the ranked features performed for identifying DSPN, the logistic regression classifier was trained with the top-1 to top-15 feature combination. Table 2 demonstrates the weighted average performance and the overall accuracies of other matrices for different models, utilizing the top-1 to top-15 features for the five-fold cross-validation through a logistic regression classifier, together with the confusion matrices for each of the cases. With more than the top-10 features, there was no major change in the performance of the logistic regression classifier. The results from the LR classifier using the top-10 ranked features for the Xgboost technique are reported in Supplementary Table S1.

The top-10-ranked features utilizing the extra tree technique have the best performance for the diagnosis of DSPN and non-DSPN patients compared to the Xgboost technique. The top-10 feature combinations provide the best performance accuracy of 92% for DSPN identification (Table 2). Although, the top-7 feature exhibits a reasonable performance in identifying DSPN and non-DSPN classes with 90% sensitivity and specificity; hence, balanced performance in identifying both classes. To establish the best feature combination between the two we considered both the top 7 and top 10 feature models.

### 3.3. Development and Evaluation of a Nomogram to Predict DSPN

Table 3 and Table 4 show the LR models for the top 7 and top 10 features, respectively. In LR models, the z-value indicates the contribution of each variable used in the model to predict the output. As seen in Table 3 and Table 4 all the features were statistically significant with a *p*-value less than 0.05. To choose the best performing model, between the top 10 and top 7 feature LR models, both models were implemented on the EDIC and independent test set from Watari et al. [28]. Table 5 shows the performance evaluation metrics for both models. The top-10 features model has an accuracy of 91% on the EDIC dataset and an accuracy of 86% on the independent dataset from Watari et al. [28] (Table 5). However, the top-7 features model exhibited consistently high and comparable performance on the EDIC and Watari et al. [28] data sets with an accuracy of 90% and 91%, respectively. Given that the LR model with the top-7 feature combination has reliable performance on both datasets we developed the nomogram and the severity grading system from the top-7 feature combinations: 10-gm filament, vibration perception (L), vibration perception (R), appearance of callus, appearance of deformities, previous diabetic neuropathy, appearance of fissure.

**Figure 3:**
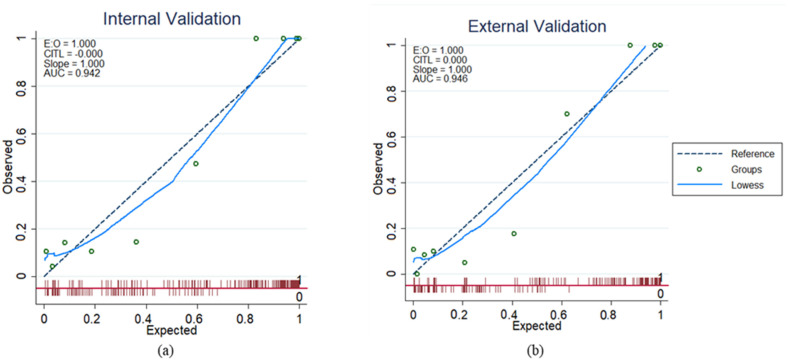
Calibration plot comparing actual and predicted DSPN probability for (**a**) the internal validation and (**b**) the external validation.

**Figure 4:**
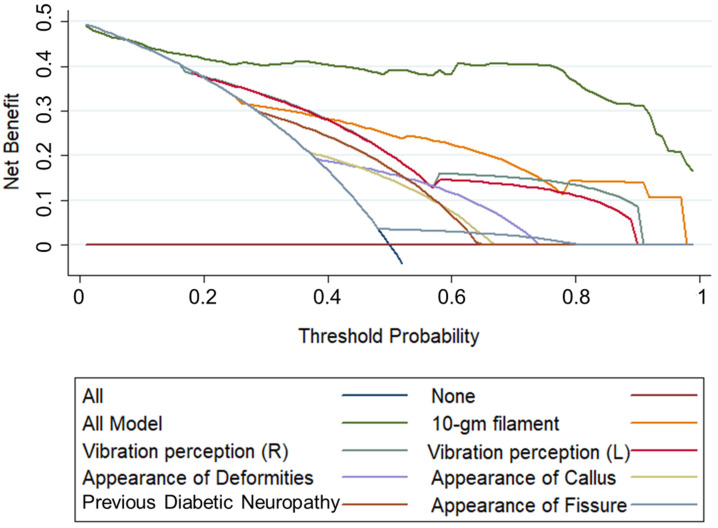
Decision curve analysis of different models for predicting DSPN severity.

Figure 3 shows the calibration plot of the training set for internal validation and the test set for external validation, with an area under the curve (AUC) of 0.94 for both, indicating good reliability of the LR model. Figure 4 illustrates the decision curve analysis comparing the net benefit of all the different models created from individual features for DSPN probability prediction. It additionally shows the performance of the overall model (all features) for DSPN probability prediction.

**Figure 5:**
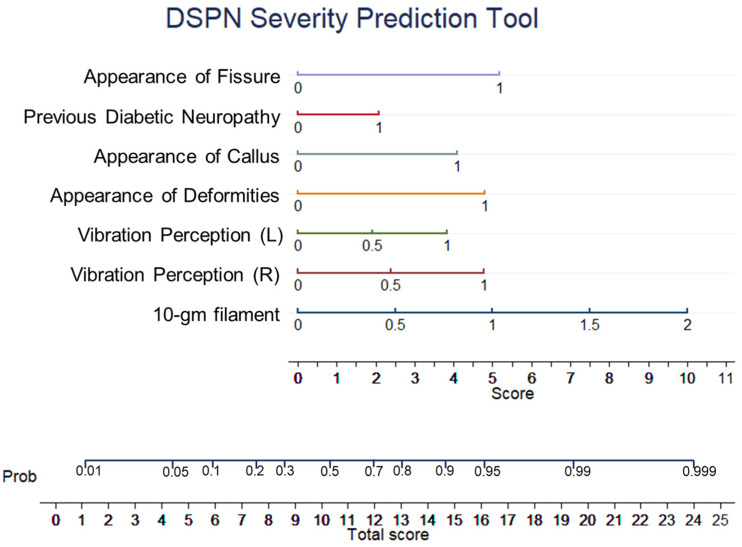
Nomogram based on multivariate logistic regression-based for probability prediction of DSPN severity. Nomogram for predicting DSPN severity was formed utilizing seven different predictors, namely 10-gm filament, Vibration perception (L), Vibration perception (R), Appearance of Callus, Appearance of Deformities, Previous Diabetic Neuropathy, Appearance of Fissure.

**Figure 6:**
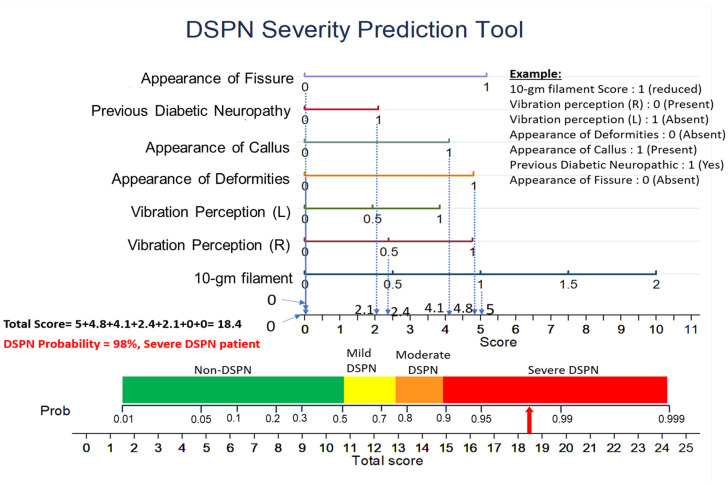
Example nomogram-based score for the probability prediction of DSPN severity.

Figure 5 shows the nomogram generated using multivariate logistic regression for DSPN probability prediction utilizing the top-7 MNSI features. The nomogram spreads over 10 rows. The top 1–7 rows represent seven MNSI variables, together with a scale indicating the corresponding responses. The eighth row is the score scale for the responses of the seven variables. Row 9 is the probability axis indicating the probability of DSPN in patients based on the MNSI responses. Row 10 is the total score scale, where all the scores for each MNSI response are added to calculate the final score. Figure 6 demonstrates an example scoring system based on a nomogram, for a DSPN patient who possesses the variable values at baseline. Individual scores for each predictor were computed and added to calculate the total score. The calculated DSPN probability is 98% and according to Table 6 the patient has severe DSPN. The DSPN probability of a patient can also be calculated using Equations (4) and (5), which were derived from the LR model for the top 7 features (Table 3).

(4) $$\begin{array}{c} Linear\;prediction\;\left(LP\right)=\left(-5.31948\right)\\ +\left(2.514831\;*\;10-\mathrm{gm}\;\mathrm{filaments}\right)\\ +\left(2.399316\;*\mathrm{Vibration}\;\mathrm{perception}\;\left(R\right)\right)\\ +\left(1.932473\;*\mathrm{Vibration}\;\mathrm{perception}\;\left(L\right)\right)\\ +\left(2.413763\;*\mathrm{Appearance}\;\mathrm{of}\;\mathrm{Deformities}\right)\\ +\left(2.064003\;*\mathrm{Appearance}\;\mathrm{of}\;\mathrm{Callus}\right)\\ +\left(1.053302\;*\mathrm{Previous}\;\mathrm{Diabetic}\;\mathrm{Neuropathy}\right)\\ +\left(2.602008\;*\;\mathrm{Appearance}\;\mathrm{of}\;\mathrm{Fissure}\right) \end{array}$$

(5) $$DSPN\;\;Probability=\frac{1}{1+e^{-LP}}$$

For each MNSI response, a score was generated by the nomogram. Supplementary Table S2 (Supplementary Materials) shows the MNSI responses and their corresponding score. All the scores corresponding to the MNSI responses were added together to obtain the total score. The total score was then used to calculate the DSPN probability from the nomogram. Using the total score and corresponding probability, we developed a four-class severity grading system as shown in Table 6. The probability values less than 50%, between 50% and 75 %, between 75% and 90%, and more than 90% were categorized into absent, mild, moderate, and severe groups, respectively.

**Table 6:** MNSI severity score from the nomogram and the corresponding severity probability of the DSPN patient.

Patient Group	Absent				Mild					Moderate				Severe			
MNSI Severity score	0	1	6.2	10.5	10.6	11.4	11.8	12.3	12.7	12.8	13.3	14	15	15.1	16.5	19	>28
DSPN Severity probability	0.5	1	10	49	50	60	65	70	74	75	80	85	90	91	95	99	99.99

### 3.4. Evaluation of Performance of the Nomogram Model

We applied the developed grading system on the train, test and independent test set and classified patients into four different classes of DSPN severity, namely absent (non-DSPN), mild, moderate and severe DSPN. For the EDIC train and test set, the patient’s severity classes were cross correlated with the EDIC binary ground truth (Table 7 and Table 8). For the EDIC training set (Table 7), out of the 1526 patients classified by the proposed grading system as absent, 89.2% were non-DSPN while the remaining 10.8% had DSPN as per the EDIC ground truth. In 292 mild DSPN patients, 55.5% were non-DSPN while the remaining 44.5% were DSPN. For patients classified as either moderate or severe, all had DSPN. With regard to the EDIC test set (Table 8), among the 635 patients classified as absent, 91.8% were non-DSPN while the remaining 8.2% had DSPN. Of the 145 patients classified as mild, 48.97% were non-DSPN while 51.03% had DSPN. Of the patients classified as either moderate or severe, all had DSPN, based on the EDIC ground truth. Finally, with regard to the independent test set (Table 9), 93.1% of the patients classified as absent were non-DSPN while the remaining 6.9% had DSPN. In patients classified as mild, there was an equal number of both DSPN and non-DSPN patients (i.e., 50% of each). For both the moderate and severe DSPN; no patient was mis-classified. Watari et al. [28] put forward a DSPN severity grading system by utilizing a fuzzy inference system (FIS) using three MNSI variables (questionnaire, vibration perception, and 10-gm monofilament) and a patient severity class using their grading system was available. In Table 10, we compare their results with our prediction models on the same MNSI data set. According to Watari et al. [28], among 102 patients, 29, 25, 27, and 21 had absent, mild, moderate, and severe DSPN, whereas based on our proposed model, 59, 10, 9 and 25 patients had absent, mild, moderate and severe DSPN (Table 10) showing a lack of agreement between the grading systems. Furthermore, according to the EDIC definition of DSPN [14,34], there were 59 non-DSPN patients and 43 DSPN patients in the study by Watari et al. [28] (Table 11). However, the fuzzy system classified 29 as non-DSPN and 73 as DSPN and as per our proposed grading system, the dataset had 58 non-DSPN and 44 DSPN patients, indicating that the proposed grading system agrees with the EDIC definition of DSPN [14,34]. However, because Watari et al. [28] selected only three variables, i.e., questionnaire, vibration perception, and tactile sensitivity for input, because the fuzzy inference system is an if/then rule-based system, there is the possibility of bias due to an inadequate number of variables for the identification of DSPN. Our prediction model could detect the moderate and severe DSPN groups accurately without any misclassification of the training, test, and independent test datasets, and additionally demonstrated better accuracy in identifying the absent DSPN class patients (Table 7, Table 8 and Table 9).

The difference in DSPN identification as per the EDIC definition and fuzzy model suggests a need to improve the latter. There was an association between different DSPN severity classes in the independent test set and the grading by Watari et al. [28]. Watari et al. [28] had 29 absent, 25 mild, 27 moderate, and 21 severe patients, whereas our model predicted 59 absent, 10 mild, 9 moderate, and 25 severe cases (Table 10) in the same groups as Watari et al. [28]. Our nomogram-based model is more robust because it considers all the important MNSI parameters in DSPN prediction and severity grading compared to only a few parameters in the fuzzy model. This scoring technique based on a nomogram can diagnose and infer the DSPN severity of patients into absent, mild, moderate, and severe (please refer to Table 6).

## 4. Discussion

Diabetic neuropathy may be classified as sensorimotor polyneuropathy or autonomic neuropathy. This research has focused on sensorimotor polyneuropathy as it has significant consequences in relation to foot ulceration, amputation and increased mortality. Whilst the ADA position statement advocates the use of symptoms, signs, and electrophysiology [1], other guidelines have suggested the use of quantitative sensory testing and intraepidermal nerve fibre density (IENFD) for diagnosing DSPN [6,7,8,9,10]. However, neurophysiology and IENFD are expensive, require specialized personnel, and are not suitable for large clinical trials. Composite screening methods that assess symptoms and signs of DSPN have been used widely [12] and include the MNSI which has been used in epidemiological studies [13,14,15,16,17], large clinical trials such as DCCT⁄EDIC [33,34,35] and the Action to Control Cardiovascular Disease in Diabetes (ACCORD) [45].

The MNSI questionnaire and examination can identify the presence of clinical neuropathy but have not been validated to grade the severity of DSPN as per the neuropathy disability score (NDS) or the neuropathy symptom score (NSS) [11,12]. Feldman et al. [13] advised that patients with a positive MNSI should undergo assessment of the Michigan diabetic neuropathy score (MDNS), which includes a clinical examination and nerve conduction studies (NCS). However, NCS have a large inter-individual variability and moderate reproducibility and are therefore not suitable for large clinical trials, unless the outcome is standardised using a central reading facility. A simple and reliable DPSN severity scoring system is highly desirable to identify patients with mild disease, in addition to those at high risk of foot ulceration.

Using a state-of-the-art machine learning model, we have designed and deployed a prediction scoring system utilizing MNSI to classify patients in the DCCT/EDIC clinical trial into absent, mild, moderate and severe DSPN. Of note, the original dataset from the EDIC clinical trial had missing and duplicate responses for many patients and therefore after eliminating duplicate samples, we imputed the dataset utilizing the MICE algorithm to predict the missing values. The MNSI variables were ranked after taking into consideration their importance index for DSPN identification using various feature ranking techniques. The extra tree algorithm was found to be the best-performing algorithm for identifying the best combination of MNSI variables. The logistic regression classifier was trained for the top 1 to 15 feature combinations using five-fold cross-validation for identifying the best combination of features. Two models with the top 7 and top 10 variables showed promising results with AUCs of 94% and 96%, respectively. The top 10 models showed better AUC, sensitivity, and accuracy compared to the top 7 ranked features model when validated on an external independent dataset by Watari et al. [28]. However, only marginal improvements were achieved by using the top-10 ranked features model from the top 7 feature model, therefore the top 7 ranked features were selected to develop the nomogram using a multivariant logistic regression model. On the basis of this nomogram, the DSPN severity grading system was proposed based on the predicted DSPN probability and total score on MNSI.

A major strength of our study is that it was undertaken using data from a large number of patients in the established DCCT/EDIC trials. Our model could infer moderate and severe DSPN without any misclassification for the train, test, and independent test set, and also exhibited high accuracy for absent DSPN. Although, misclassification was evident for those with mild DSPN, using MNSI as ground truth may not be adequate as it relies primarily on identifying large fibre damage with the possibility of missing earlier small fibre damage evident in mild DSPN. Furthermore, the model performed well in patients with either type 1 or type 2 diabetes. However, this model has only been validated with the performance of the FIS model used by Watari et al. [28]. In the future, we plan to validate the model performance utilising NCS and NDS to improve reproducibility and robustness of the model. In conclusion, we have designed, implemented and validate a DSPN severity scoring system based on a machine learning model, utilizing MNSI which could aid researchers and clinicians as an auxiliary decision-making system. This study highlights the potential for machine learning-based applications to diagnose and stage DSPN severity.

## 5. Conclusions

The detection of early DSPN is key to preventing foot ulceration, amputation and increased mortality in patients with diabetes. MNSI, originally developed to screen for DSPN, has been used widely in epidemiological studies and even in clinical trials, even though it lacks a severity grading system. In this study, we have applied ML-based approaches to develop a DSPN severity grading system for MNSI. Using the extra tree feature ranking technique, we have identified the seven best MNSI features i.e., vibration perception (R), 10-gm monofilament, presence of diabetic neuropathy, vibration perception (L), the appearance of callus, deformities and fissure for identifying DSPN. These features were used to develop a nomogram-based probability model, and from the probability model, a severity scoring technique was proposed and validated in three data sets. MNSI could therefore be easily used to detect DSPN severity in large clinical trials.

## Figures and Tables

**Table 1:** EDIC patient baseline characteristics.

N: 1341 M: 658 (52.39%) F: 598 (47.61%)	Mean	Std. Error of Mean	Minimum	Maximum
Age (years)	35.98 ± 6.95	0.19	20.16	50.99
HbA1_C_ (%)	8.23 ± 1.39	0.04	0.00	14.00
BMI (kg/m^2^)	26.24 ± 4.16	0.11	0.00	49.82
Diabetes duration (years)	14.55 ± 4.91	0.13	0.00	27.00
HDL Cholesterol (mg/dL)	52.69 ± 16.05	0.44	0.00	121.00
LDL Cholesterol (mg/dL)	110.75 ± 36.33	0.99	0.00	280.00

**Table 2:** Average performance matrix vs. confusion matrix from five-fold cross-validation for top-15-ranked features utilizing the MICE data imputation and logistic regression classification techniques for extra tree feature selection algorithms.

	Sensitivity (%)	Specificity (%)	Accuracy (%)	Precision (%)	F1 Score (%)	Non-DSPN		DSPN	
						TN	FP	FN	TP
Top 1 Feature	72 ± 0.02	80 ± 0.04	76 ± 0.02	78 ± 0.03	75 ± 0.02	1732	445	619	1558
Top 2 Features	73 ± 0.05	85 ± 0.05	79 ± 0.02	83 ± 0.04	77 ± 0.02	1845	332	591	1586
Top 3 Features	75 ± 0.03	86 ± 0.04	80 ± 0.01	84 ± 0.03	79 ± 0.01	1869	308	548	1629
Top 4 Features	77 ± 0.04	86 ± 0.02	82 ± 0.03	85 ± 0.03	81 ± 0.03	1877	300	496	1681
Top 5 Features	86 ± 0.02	92 ± 0.03	89 ± 0.02	91 ± 0.03	88 ± 0.02	1994	183	315	1862
Top 6 Features	88 ± 0.03	86 ± 0.04	87 ± 0.03	87 ± 0.03	87 ± 0.03	1879	298	260	1917
Top 7 Features	90 ± 0.02	90 ± 0.03	90 ± 0.02	90 ± 0.02	90 ± 0.02	1954	223	220	1957
Top 8 Features	89 ± 0.02	92 ± 0.04	90 ± 0.02	91 ± 0.04	90 ± 0.02	1995	182	238	1939
Top 9 Features	89 ± 0.03	90 ± 0.07	89 ± 0.03	90 ± 0.06	89 ± 0.03	1949	228	233	1944
Top 10 Features	92 ± 0.01	93 ± 0.05	92 ± 0.02	93 ± 0.05	92 ± 0.02	2019	158	185	1992
Top 11 Features	91 ± 0.01	92 ± 0.05	92 ± 0.03	92 ± 0.05	91 ± 0.03	2001	176	194	1983
Top 12 Features	91 ± 0.02	92 ± 0.05	92 ± 0.03	92 ± 0.05	91 ± 0.03	2003	174	195	1982
Top 13 Features	91 ± 0.02	92 ± 0.04	92 ± 0.02	92 ± 0.03	91 ± 0.02	2012	165	204	1973
Top 14 Features	90 ± 0.02	92 ± 0.05	91 ± 0.03	92 ± 0.05	91 ± 0.02	2007	170	210	1967
Top 15 Features	90 ± 0.02	92 ± 0.06	91 ± 0.04	92 ± 0.06	91 ± 0.04	2008	169	209	1968

**Table 3:** Logistic regression analysis for constructing the nomogram to predict DSPN utilizing the top 7 variables by the extra tree feature ranking technique.

Outcome	Coef.	Std. Err.	z	P > z	[95% Conf. Interval]	
10-gm filament	2.514831	0.137814	18.25	0.00	2.24472	2.784941
Vibration perception (R)	2.399316	0.249416	9.62	0.00	1.91047	2.888162
Vibration perception (L)	1.932473	0.247976	7.79	0.00	1.446448	2.418498
Appearance of Deformities	2.413763	0.142204	16.97	0.00	2.135049	2.692477
Appearance of Callus	2.064003	0.13319	15.5	0.00	1.802955	2.325051
Previous Diabetic Neuropathy	1.053302	0.125036	8.42	0.00	0.808235	1.298369
Appearance of Fissure	2.602008	0.272765	9.54	0.00	2.067398	3.136619
_cons	−5.31948	0.207402	−25.65	0.00	−5.72598	−4.91298

**Table 4:** Logistic regression analysis for constructing the nomogram for predicting DSPN utilizing the top 10 variables by the extra tree feature ranking technique.

Outcome	Coef.	Std. Err.	z	P > z	[95% Conf. Interval]	
10-gm filament	3.084504	0.1696	18.19	0.00	2.752094	3.416913
Vibration perception (R)	3.003988	0.285598	10.52	0.00	2.444225	3.56375
Vibration perception (L)	2.326558	0.282243	8.24	0.00	1.773372	2.879744
Appearance of Deformities	3.202711	0.176598	18.14	0.00	2.856585	3.548837
Appearance of Callus	2.886776	0.169801	17	0.00	2.553974	3.219579
Previous Diabetic Neuropathy	0.634693	0.140511	4.52	0.00	0.359297	0.910089
Appearance of Fissure	3.52151	0.309166	11.39	0.00	2.915556	4.127464
Numb Leg	0.941649	0.149556	6.3	0.00	0.648525	1.234772
Burning Leg	1.235312	0.153058	8.07	0.00	0.935324	1.535301
Bed Cover Touch	2.655393	0.244644	10.85	0.00	2.175899	3.134887
_cons	−7.49854	0.306272	−24.48	0.00	−8.09883	−6.89826

**Table 5:** Evaluation of the performance for the top 7 and 10 features using a logistic regression model to construct the nomogram for DSPN prediction.

Prediction Model	Test Sets	Sensitivity (%)	Specificity (%)	Accuracy (%)	Precision (%)	F1 Score (%)	Confusion Matrix			
							Non-DSPN		DSPN	
							TN	FP	FN	TP
Top 7 Variable model	EDIC Test Set	91	89	90	86	88	583	71	44	429
	Independent Test Set	91	92	91	89	90	54	5	4	39
Top 10 Variable model	EDIC Test Set	91	92	91	89	90	598	56	42	431
	Independent Test Set	93	81	86	78	85	48	11	3	40

True positive (TP): True DSPN patients. True negative (TN): True Non-DSPN. False-positive (FP): Non-DSPN patients, classified as DSPN patients. False-negative (FN): DSPN patients, classified as non-DSPN patients.

**Table 7:** The association among different DSPN severity groups and the actual outcomes in the EDIC training dataset, utilizing the Fisher exact probability test.

DSPN Severity Class	Outcome		Total
	Non-DSPN	DSPN	
Absent	1361 (89.2%)	165(10.8%)	1526 (100%)
Mild	162 (55.5%)	130 (44.5%)	292 (100%)
Moderate	0 (0%)	282 (100%)	282 (100%)
Severe	0 (0%)	947 (100%)	947 (100%)
Total	1523 (50%)	1524 (50%)	3047 (100%)

**Table 8:** The association among different DSPN severity groups and the actual outcomes in the EDIC testing dataset, utilizing the Fisher exact probability test.

DSPN Severity Class	Outcome		Total
	Non-DSPN	DSPN	
Absent	583 (91.8%)	52 (8.2%)	635
Mild	71(48.97%)	74 (51.03%)	145
Moderate	0 (0%)	120 (100%)	120
Severe	0 (0%)	407 (100%)	407
Total	654 (50%)	653 (50%)	1307

**Table 9:** The association among different DSPN severity groups and actual outcomes in the independent test dataset from Watari et al. [28], using Fisher exact probability test.

DSPN Severity Class	Outcome		Total
	Non-DSPN	DSPN	
Absent	54 (93.1%)	4 (6.9%)	58 (100%)
Mild	5 (50%)	5 (50%)	10 (100%)
Moderate	0 (0%)	9 (100%)	9 (100%)
Severe	0 (0%)	25 (100%)	25 (100%)
Total	59 (57.8%)	43 (42.2%)	102 (100%)

**Table 10:** The association among different DSPN severity groups and severity grading by Watari et al. [28] in the independent test dataset using Fisher exact probability test.

DSPN Severity Grading by Our Model	DSPN Severity Grading by Watari et al., 2014 [28]				Total
	Absent	Mild	Moderate	Severe	
Absent	28	18	12	0	59
Mild	0	2	5	3	10
Moderate	1	3	3	2	9
Severe	0	2	7	16	25
Total	29	25	27	21	102

**Table 11:** Performance comparison of our proposed MNSI cut-offs for binary classification on an independent test cohort (Watari et al., 2014 [28]).

MNSI Cut-Off	Non-DSPN	DSPN
Feldman et al. [10]	59	43
Watari et al. [28]	29	73
Our Prediction Model	58	44

## Data Availability

The datasets used in this study is not publicly available.

## Supplementary Materials

The following supporting information can be downloaded at: https://www.mdpi.com/article/10.3390/diagnostics13020264/s1. Figure S1. Top-ranked 10 features identified using Random Forest algorithm from data imputed using MICE algorithm. Figure S2. Top-ranked 10 features identified using Xgboost algorithm from data imputed using MICE algorithm. Table S1. Comparison of the average performance matrix and confusion matrix from five-fold cross-validation for top 1 to 10 features using MICE data imputation and logistic regression classification techniques for XGBoost feature selection algorithms. Table S2. MNSI variables score from the generated nomogram.

## References

[1] Pop-Busui R., Boulton A.J.M., Feldman E.L., Bril V., Freeman R., Malik R.A., Sosenko J.M., Ziegler D. (2017). Diabetic Neuropathy: A Position Statement by the American Diabetes Association. Diabetes Care.

[2] Malik R.A. (2014). Which Test for Diagnosing Early Human Diabetic Neuropathy?. Diabetes.

[3] Malik R.A., Williamson S., Abbott C., Carrington A.L., Iqbal J., Schady W., Boulton A.J.M. (1998). Effect of Angiotensin-Converting-Enzyme (ACE) Inhibitor Trandolapril on Human Diabetic Neuropathy: Randomised Double-Blind Controlled Trial. Lancet.

[4] Malik R.A., Tesfaye S., Ziegler D. (2013). Medical Strategies to Reduce Amputation in Patients with Type 2 Diabetes. Diabet. Med..

[5] Haque F., Reaz M.B.I., Ali S.H., Arsad N., Enamul M., Chowdhury H. (2020). Performance Analysis of Noninvasive Electrophysiological Methods for the Assessment of Diabetic Sensorimotor Polyneuropathy in Clinical Research: A Systematic Review and Meta-Analysis with Trial Sequential Analysis. Sci. Rep..

[6] Tesfaye S., Boulton A.J.M., Dyck P.J., Freeman R., Horowitz M., Kempler P., Lauria G., Malik R.A., Spallone V., Vinik A. (2010). Diabetic Neuropathies: Update on Definitions, Diagnostic Criteria, Estimation of Severity, and Treatments. Diabetes Care.

[7] Taksandea B., Ansaria S., Jaikishana A., Karwasara V. (2011). The Diagnostic Sensitivity, Specificity and Reproducibility of the Clinical Physical Examination Signs in Patients of Diabetes Mellitus for Making Diagnosis of Peripheral Neuropathy. J. Endocrinol. Metab..

[8] Perkins B., Bril V. (2014). Electrophysiologic Testing in Diabetic Neuropathy.

[9] Ahmed A., Bril V., Orszag A., Paulson J., Yeung E., Ngo M., Orlov S., Perkins B.A. (2012). Detection of Diabetic Sensorimotor Polyneuropathy by Corneal Confocal Microscopy in Type 1 Diabetes: A Concurrent Validity Study. Diabetes Care.

[10] Sacco I.C., Akashi P.M., Hennig E.M. (2010). A Comparison of Lower Limb EMG and Ground Reaction Forces between Barefoot and Shod Gait in Participants with Diabetic Neuropathic and Healthy Controls. BMC Musculoskelet. Disord..

[11] Won J.C., Park T.S. (2016). Recent Advances in Diagnostic Strategies for Diabetic Peripheral Neuropathy. Endocrinol. Metab..

[12] Atre J., Ganvir S. (2019). Screening Instrument for Clinical Diagnosis of Peripheral Neuropathy in Diabetes—A Review. Indian J. Physiother. Occup. Ther.—Int. J..

[13] Feldman E.L., Stevens M.J., Thomas P.K., Brown M.B., Canal N., Greene D.A. (1994). A Practical Two-Step Quantitative Clinical and Electrophysiological Assessment for the Diagnosis and Staging of Diabetic Neuropathy. Diabetes Care.

[14] Martin C.L., Albers J., Herman W.H., Cleary P., Waberski B., Greene D.A., Stevens M.J., Feldman E.L. (2006). Neuropathy among the Diabetes Control and Complications Trial Cohort 8 Years after Trial Completion. Diabetes Care.

[15] Qureshi M.S., Iqbal M., Zahoor S., Ali J., Javed M.U. (2017). Ambulatory Screening of Diabetic Neuropathy and Predictors of Its Severity in Outpatient Settings. J. Endocrinol. Investig..

[16] Andersen S.T., Witte D.R., Dalsgaard E.M., Andersen H., Nawroth P., Fleming T., Jensen T.S.M., Finnerup N.B., Jensen T.S.M., Lauritzen T. (2018). Risk Factors for Incident Diabetic Polyneuropathy in a Cohort with Screen-Detected Type 2 Diabetes Followed for 13 Years: Addition-Denmark. Diabetes Care.

[17] Christensen D.H., Knudsen S.T., Gylfadottir S.S., Christensen L.B., Nielsen J.S., Beck-Nielsen H., Sørensen H.T., Andersen H., Callaghan B.C., Feldman E.L. (2020). Metabolic Factors, Lifestyle Habits, and Possible Polyneuropathy in Early Type 2 Diabetes: A Nationwide Study of 5,249 Patients in the Danish Centre for Strategic Research in Type 2 Diabetes (Dd2) Cohort. Diabetes Care.

[18] Moghtaderi A., Bakhshipour A., Rashidi H. (2006). Validation of Michigan Neuropathy Screening Instrument for Diabetic Peripheral Neuropathy. Clin. Neurol. Neurosurg..

[19] Herman W.H., Pop-Busui R., Braffett B.H., Martin C.L., Cleary P.A., Albers J.W., Feldman E.L. (2012). Use of the Michigan Neuropathy Screening Instrument as a Measure of Distal Symmetrical Peripheral Neuropathy in Type1 Diabetes: Results from the Diabetes Control and Complications Trial/Epidemiology of Diabetes Interventions and Complications. Diabet. Med..

[20] Pabellano-Tiongson M.L.G.P., Javelosa G.F.J., Tan A.D.A. (2018). The Validity of the Filipino Version of the Michigan Neuropathy Screening Instrument as a Measure of Distal Symmetric Peripheral Neuropathy among Diabetic Patients at the Uermmmci Outpatient Deparment. Asian J. Res. Rep. Neurol..

[21] Park J.H., Kim D.S. (2018). The Necessity of the Simple Tests for Diabetic Peripheral Neuropathy in Type 2 Diabetes Mellitus Patients without Neuropathic Symptoms in Clinical Practice. Diabetes Metab. J..

[22] Haque F., Reaz M.B.I., Chowdhury M.E.H., Ezeddin M., Kiranyaz S., Alhatou M., Ali S.H.M., Bakar A.A.A., Srivastava G. (2022). Machine Learning-Based Diabetic Neuropathy and Previous Foot Ulceration Patients Detection Using Electromyography and Ground Reaction Forces during Gait. Sensors.

[23] Haque F., Reaz M.B.I., Chowdhury M.E.H., Kiranyaz S., Ali S.H.M., Alhatou M., Habib R., Bakar A.A.A., Arsad N., Srivastava G. (2022). Performance Analysis of Conventional Machine Learning Algorithms for Diabetic Sensorimotor Polyneuropathy Severity Classification Using Nerve Conduction Studies. Comput. Intell. Neurosci..

[24] Haque F., Reaz M.B.I., Chowdhury M.E.H., Srivastava G., Ali S.H.M., Bakar A.A.A., Bhuiyan M.A.S. (2021). Performance Analysis of Conventional Machine Learning Algorithms for Diabetic Sensorimotor Polyneuropathy Severity Classification. Diagnostics.

[25] Thorsen-Meyer H.C., Nielsen A.B., Nielsen A.P., Kaas-Hansen B.S., Toft P., Schierbeck J., Strøm T., Chmura P.J., Heimann M., Dybdahl L. (2020). Dynamic and Explainable Machine Learning Prediction of Mortality in Patients in the Intensive Care Unit: A Retrospective Study of High-Frequency Data in Electronic Patient Records. Lancet Digit. Health.

[26] Zhao X., Zhang X., Ran X., Xu Z., Ji L. (2020). Simple-to-Use Nomogram for Evaluating the Incident Risk of Moderate-to-Severe LEAD in Adults with Type 2 Diabetes: A Cross-Sectional Study in a Chinese Population. Sci. Rep..

[27] Picon A.A.P., Ortega N.R.S.N., Watari R., Sartor C., Sacco I.C.N.I. (2012). Classification of the Severity of Diabetic Neuropathy: A New Approach Taking Uncertainties into Account Using Fuzzy Logic. Clinics.

[28] Watari R., Sartor C.D., Picon A.P., Butugan M.K., Amorim C.F., Ortega N.R.S., Sacco I.C.N. (2014). Effect of Diabetic Neuropathy Severity Classified by a Fuzzy Model in Muscle Dynamics during Gait. J. NeuroEng. Rehabil..

[29] Kazemi M., Moghimbeigi A., Kiani J., Mahjub H., Faradmal J. (2016). Diabetic Peripheral Neuropathy Class Prediction by Multicategory Support Vector Machine Model: A Cross-Sectional Study. Epidemiol. Health.

[30] Haque F., Reaz M.B.I., Chowdhury M.E.H., Hashim F.H., Arsad N., Ali S.H.M. (2021). Diabetic Sensorimotor Polyneuropathy Severity Classification Using Adaptive Neuro-Fuzzy Inference System. IEEE Access.

[31] Reddy S., Mahesh G., Preethi N. (2018). Evolving A Neural Network to Predict Diabetic Neuropathy. ICST Trans. Scalable Inf. Syst..

[32] Chen S., Kang L., Lu Y., Wang N., Lu Y., Lo B., Yang G.Z. Discriminative Information Added by Wearable Sensors for Early Screening—A Case Study on Diabetic Peripheral Neuropathy. Proceedings of the 2019 IEEE 16th International Conference on Wearable and Implantable Body Sensor Networks, BSN 2019—Proceedings.

[33] (1999). Epidemiology of Diabetes Interventions and Complications (EDIC) Research Group Epidemiology of Diabetes Interventions and Complications (EDIC): Design, Implementation, and Preliminary Results of a Long-Term Follow-up of the Diabetes Control and Complications Trial Cohort. Diabetes Care.

[34] Pop-Busui R., Herman W.H., Feldman E.L., Low P.A., Martin C.L., Cleary P.A., Waberski B.H., Lachin J.M., Albers J.W. (2010). DCCT and EDIC Studies in Type 1 Diabetes: Lessons for Diabetic Neuropathy Regarding Metabolic Memory and Natural History. Curr. Diabetes Rep..

[35] Martin C.L., Albers J.W., Pop-Busui R. (2014). Neuropathy and Related Findings in the Diabetes Control and Complications Trial/Epidemiology of Diabetes Interventions and Complications Study. Diabetes Care.

[36] Wulff J.N., Ejlskov L. (2017). Multiple Imputation by Chained Equations in Praxis: Guidelines and Review. Electron. J. Bus. Res. Methods.

[37] Austin P.C., White I.R., Lee D.S., van Buuren S. (2020). Missing Data in Clinical Research: A Tutorial on Multiple Imputation. Can. J. Cardiol..

[38] Hegde H., Shimpi N., Panny A., Glurich I., Christie P., Acharya A. (2019). MICE vs PPCA: Missing Data Imputation in Healthcare. Inform. Med. Unlocked.

[39] Breiman L. (2001). Random Forests. Mach. Learn..

[40] Chen T., Guestrin C. XGBoost: A Scalable Tree Boosting System. Proceedings of the KDD ’16.

[41] Geurts P., Ernst D., Wehenkel L. (2006). Extremely Randomized Trees. Mach. Learn..

[42] Le Cessie S., van Houwelingen J.C. (1992). Ridge Estimators in Logistic Regression. Appl. Stat..

[43] Tolles J., Meurer W.J. (2016). Logistic Regression Relating Patient Characteristics to Outcomes JAMA Guide to Statistics and Methods. JAMA.

[44] Zlotnik A., Abraira V. (2015). A General-Purpose Nomogram Generator for Predictive Logistic Regression Models. Stata J..

[45] Pop-Busui R., Evans G.W., Gerstein H.C., Fonseca V., Fleg J.L., Hoogwerf B.J., Genuth S., Grimm R.H., Corson M.A., Prineas R. (2010). Effects of Cardiac Autonomic Dysfunction on Mortality Risk in the Action to Control Cardiovascular Risk in Diabetes (ACCORD) Trial. Diabetes Care.

