# Supplementary material for: A Machine Learning-Based Severity Prediction Tool for the Michigan Neuropathy Screening Instrument
Source: Diagnostics (Basel). 2023 Jan 11;13(2):264. doi: 10.3390/diagnostics13020264 (PMC9857736; doi:10.3390/diagnostics13020264)
Supplement: Supplementary file 1 [file diagnostics-13-00264-s001.zip › diagnostics-2001927-supplementary.pdf]

A Machine Learning-Based Severity Prediction Tool for Michigan Neuropathy Screening Instrumentations

Fahmida Haque [1,2], Mamun B. I. Reaz [1,3,*], Muhammad E. H. Chowdhury [4,*], Mohd Ibrahim bin Shapiai @ Abd. Razak [3], Rayaz Malik [5], Mohammed Alhatou [6], Syoji Kobashi [7], Iffat Ara [4], Sawal H. M. Ali [1], Ahmad A. A. Bakar [1] and Mohammad A. S. Bhuiyan [8,*]

[1] Centre of Advanced Electronic and Communication Engineering, Department of Electrical, Electronic and Systems Engineering, Universiti Kebangsaan Malaysia, 43600 UKM, Bangi, Selangor, Malaysia

[2] Laboratory of Emotions Neurobiology, Nencki Institute of Experimental Biology, Polish Academy of Sciences, Ludwika Pasteura 3, 02-093 Warszawa, Poland

[3] Malaysia-Japan International Institute of Technology, Universiti Teknologi Malaysia, Jalan Sultan Yahya Petra, 54100 Kuala Lumpur, Malaysia

[4] Department of Electrical Engineering, Qatar University, Doha 2713, Qatar

[5] Department of Medicine, Weill Cornell Medicine-Qatar, Ar-Rayyan 24144, Qatar

[6] Neuromuscular Division, Hamad General Hospital, Doha 3050, Qatar

[7] Department of Neurology, Al khor Hospital, Doha 3050, Qatar

[8] Graduate School of Engineering, University of Hyogo, Himeji 678-1297, Hyogo, Japan

[9] Department of Electrical and Electronics Engineering, Xiamen University Malaysia, Sepang 43900, Malaysia

* Correspondence: mamun@ukm.edu.my (M.B.I.R.); mchowdhury@qu.edu.qa (M.E.H.C.); arifsobhan.bhuiyan@xmu.edu.my (M.A.S.B.)

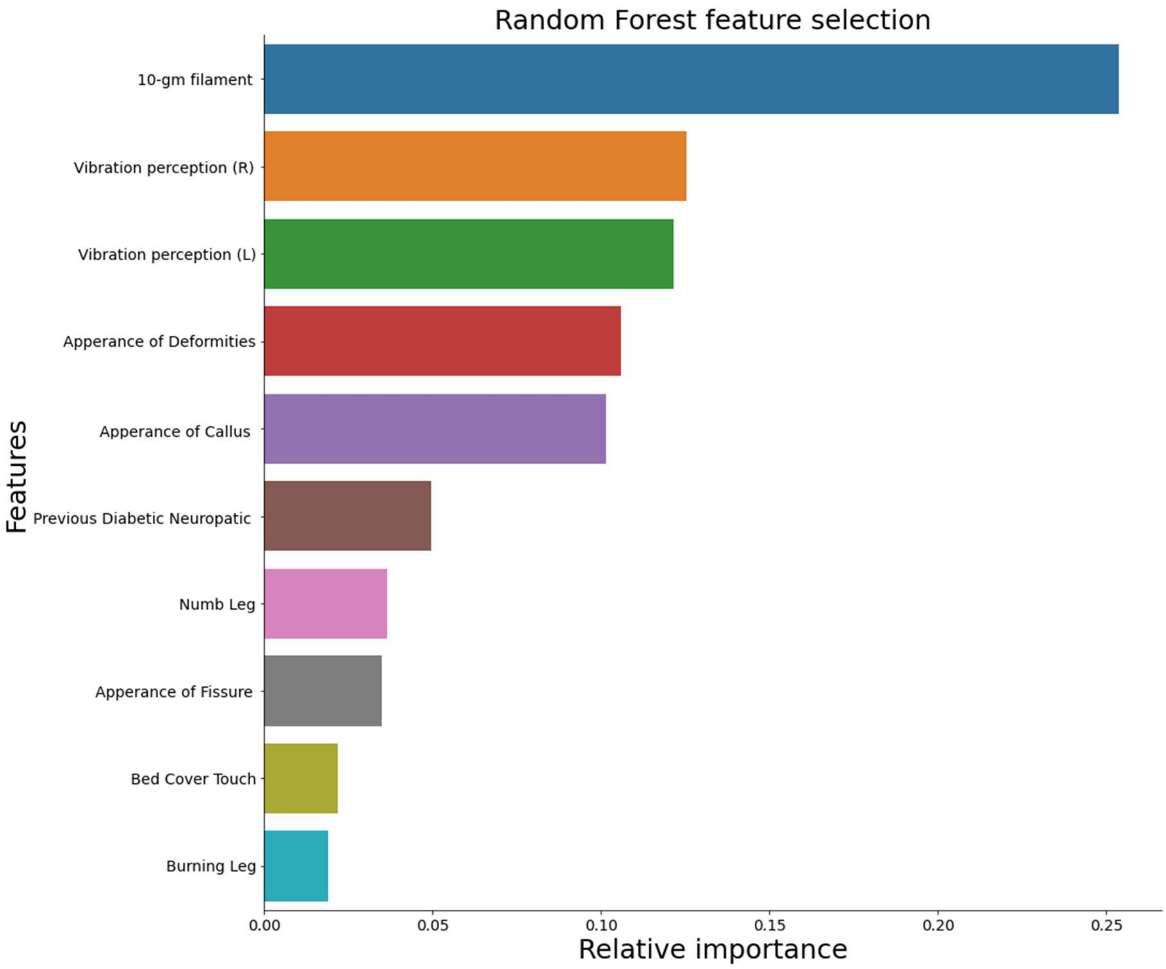

Figure S1. Top-ranked 10 features identified using Random Forest algorithm from data imputed using MICE algorithm.

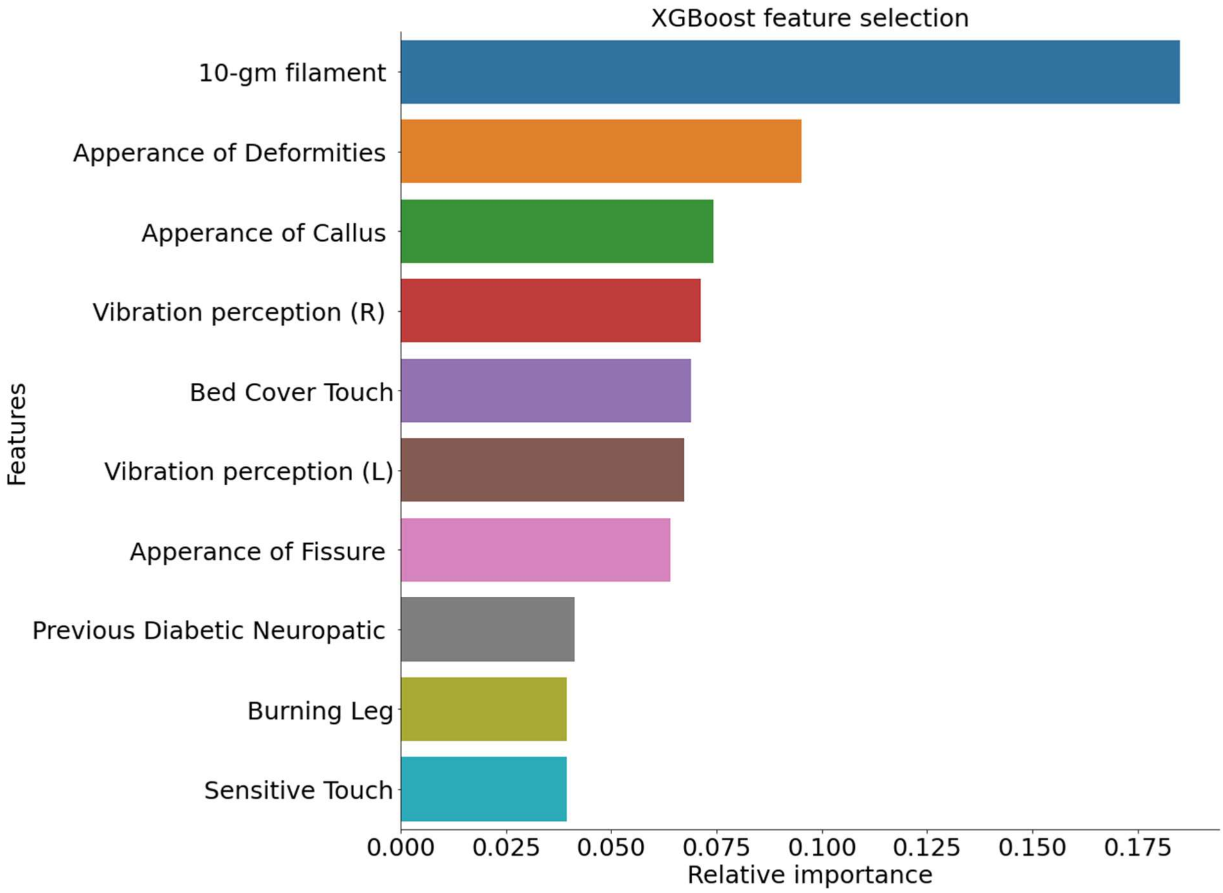

Figure S2. Top-ranked 10 features identified using Xgboost algorithm from data imputed using MICE algorithm

Table S1. Comparison of the average performance matrix and confusion matrix from five-fold cross-validation for top 1 to 10 features using MICE data imputation and logistic regression classification techniques for XGBoost feature selection algorithms.

	Sensitivity	Specificity	Accuracy	Precision	F1 Score	Non-DSPN		DSPN	
						TP	FN	FP	TN
Top 1 Feature	0.61±0.11	0.89±0.03	0.78±0.05	0.81±0.05	0.70±0.09	1975	236	625	995
Top 2 Features	0.65±0.10	0.88±0.02	0.78±0.05	0.80±0.05	0.72±0.08	1947	264	560	1060
Top 3 Features	0.75±0.05	0.91±0.03	0.84±0.03	0.86±0.05	0.80±0.04	2022	189	410	1210
Top 4 Features	0.79±0.03	0.90±0.07	0.85±0.05	0.85±0.09	0.82±0.05	1981	230	342	1278
Top 5 Features	0.80±0.05	0.89±0.07	0.85±0.06	0.84±0.09	0.82±0.06	1969	242	320	1300
Top 6 Features	0.85±0.04	0.96±0.04	0.91±0.03	0.94±0.06	0.89±0.04	2118	93	241	1379
Top 7 Features	0.90±0.03	0.94±0.03	0.91±0.02	0.94±0.04	0.90±0.03	2057	154	162	1458
Top 8 Features	0.91±0.04	0.95±0.04	0.93±0.03	0.93±0.06	0.92±0.04	2099	112	150	1470
Top 9 Features	0.89±0.04	0.91±0.04	0.90±0.03	0.90±0.06	0.89±0.04	2014	197	187	1433
Top 10 Features	0.88±0.05	0.94±0.04	0.91±0.03	0.91±0.05	0.90±0.04	2073	138	191	1429

Table S2. MNSI variables score from the generated nomogram.

Top 7 MNSI variables	Response on MNSI	Corresponding Score
10-gm filament	0	0
	0.5	2.5
	1	5
	1.5	7.5
	2	10
Vibration perception (R)	0	0
	0.5	2.4
	1	4.8
Vibration perception (L)	0	0
	0.5	1.9
	1	3.8
Appearance of Deformities	0	0
	1	4.8
Appearance of Callus	0	0
	1	4.1
Previous Diabetic Neuropathic	0	0
	1	2.1
Appearance of Fissure	0	0
	1	5.2
